# Aspirin and Its Potential Preventive Role in Cancer: An Umbrella Review

**DOI:** 10.3389/fendo.2020.00003

**Published:** 2020-01-23

**Authors:** Yongxi Song, Xi Zhong, Peng Gao, Cen Zhou, Jinxin Shi, Zhonghua Wu, Zhexu Guo, Zhenning Wang

**Affiliations:** Key Laboratory of Precision Diagnosis and Treatment of Gastrointestinal Tumors, Ministry of Education, Department of Surgical Oncology and General Surgery, The First Affiliated Hospital of China Medical University, Shenyang, China

**Keywords:** aspirin, cancer, risk, preventive, umbrella review

## Abstract

**Background:** Aspirin is one of the most commonly prescribed drugs worldwide and has been reported to possess anti-cancer properties in addition to antipyretic and analgesic effects. This umbrella review summarizes systematic reviews and meta-analyses that investigate the association between aspirin and cancer risk, aiming to help clinical and public health decision-makers interpret the results of these studies when re-positioning aspirin.

**Methods:** An umbrella review of systematic reviews and meta-analyses.

**Results:** The associations that reached statistical significance (17 in total) indicated potential preventive effects of aspirin on certain cancers or precancerous lesions. We found that no association was supported by strong evidence. Only one association (aspirin and overall cancer risk) was supported by highly suggestive evidence. The evidence supporting the association between aspirin and the risk of breast cancer, non-cardia gastric cancer, or prostate cancer was considered to be highly suggestive. The remaining 23 associations were supported by weak (13) or not suggestive evidence (10).

**Conclusions:** The association between aspirin and a reduced risk of esophageal squamous cell carcinoma is supported by strong evidence, researchers and policy makers should pay more attention to the potential merit of repositioning aspirin to prevent esophageal squamous cell carcinoma.

## Introduction

Cancer is a worldwide life-threatening public health problem, with ~40% of the population in developed countries suffering from cancer during their lifetime, with the cancer risk in developing countries also increasing (1). To better cope with the crisis of the global cancer burden, the concept of drug repositioning has been introduced in the anti-cancer field (2). Several successful examples such as metformin (3), digoxin (4), and thalidomide (5) show promising prospects in repositioning non-cancer drugs to prevent or treat cancers.

Aspirin, a widely-prescribed non-steroidal anti-inflammatory drug (NSAID) with antipyretic and analgesic effects, was reported to reduce the incidence of colorectal cancer (CRC) and colorectal adenoma in the systematic review and meta-analysis prepared for the U.S. Preventive Services Task Force (USPSTF) in 2007 (6). However, on account of the severe side effects of aspirin including gastrointestinal and cerebral hemorrhage, the USPSTF recommended against use of aspirin to prevent CRC in adults at average risk for CRC at that time (7). More recently, in 2016, based on the finding that the risk for CRC in adults decreases after 5–10 years of daily use of aspirin, the USPSTF updated their recommendations and claimed that adults aged 50–59 years with a life expectancy of more than 10 years should use aspirin as a prevention for CRC if they are not facing increased risk for bleeding and are willing to receive daily use of aspirin for at least 10 years (8). The established effect of aspirin on reducing the risk of CRC inspired and facilitated numerous studies on the potential preventive role of aspirin on other cancers (9, 10), contributing to the publication of several relevant systematic reviews and meta-analyses (11–13). However, clinicians and policy makers are overwhelmed with the number of these studies and concerned with the validity of current evidence in this field due to the substantial heterogeneity and potential bias in these systematic reviews (14).

This umbrella review aims to systematically synthesize knowledge from previously published systematic reviews and meta-analyses exploring the potential preventive role of aspirin on various cancers, thus providing a bird's-eye view of the current highest level of evidence in this field which may help clinicians, public health professionals, and policy makers interpret the results (15). For clarification, considering that the evidence is strong for the use of aspirin in the prevention of CRC or colorectal adenoma and CRC-specific studies have not been covered in great depth in this review.

## Methods

### Search Strategy and Eligibility Criteria

PubMed and Embase were systematically searched to identify systematic reviews and meta-analyses, published up to September 2nd, 2018, on the association between aspirin use and cancer risk. We also conducted manual screening of references to identify relevant studies. The following terms were used: (aspirin OR NSAIDS OR non-steroidal anti-inflammatory drugs) AND (cancer OR tumor OR malignancy OR neoplasm) AND (meta-analysis OR systematic review). Potentially relevant articles were selected after title and abstract screening, and eligible articles were included after full-text review. The study selection was independently conducted by two authors.

The criteria for eligibility were: (1) systematic reviews and meta-analyses on the associations between aspirin use and cancer incidence; (2) studies investigating the incidence of the same cancer in different populations; and (3) studies focusing on the subtypes of a specific cancer. The largest study was selected when more than one study investigated the same associations.

### Data Extraction

The first author; year of publication; cancer type; number of included studies; number of cases and subjects; relative risk estimates, including risk ratio (RR) and odds ratio (OR) and the corresponding 95% confidence interval (CI) were retrieved from the included systematic reviews and meta-analyses. For primary studies from each systematic review and meta-analysis included, the first author, number of cases and subjects, and relative risk estimates (RR and OR) and the corresponding 95% CI were extracted for further analysis. Data were extracted independently by two authors, and any divergences were resolved by consensus.

### Quality Assessment

The methodological quality of the eligible systematic reviews and meta-analyses was evaluated independently by two authors using AMSTAR (A MeaSurement Tool to Assess systematic Reviews) version 2.0. Discrepancies were resolved through discussion. AMSTAR 2.0 measures 16 items, allows a more comprehensive evaluation of systematic reviews, and focuses more on the systematic reviews that include non-randomized studies compared with AMSTAR (11 items) (16). In addition, AMSTAR 2.0 rates the methodological quality of the review as high, moderate, low, or critically low instead of creating an overall score.

### Statistical Analysis

Estimation of the summary effect—for each association between aspirin and cancer risk, a random-effect model was chosen to quantitatively synthesize the relative risk estimates and the 95% CI and calculate the corresponding *P*-value for the summary effect (17).

Assessment of heterogeneity—Cochran's Q test and the I^2^ statistic were used to assess heterogeneity among studies. We also calculated the 95% CI of I^2^ to evaluate the uncertainty around heterogeneity estimates (18).

Estimation of prediction intervals−95% prediction intervals (PI) were calculated to predict the potential preventive role of aspirin in an individual study setting and were more conservative than the overall effect indicated by 95% CI (19).

Evaluation of small-study effects—we performed Egger's regression asymmetry test to identify small-study effects, which indicate publication bias, chance, or genuine heterogeneity (20). A *P*-value smaller than 0.10 was chosen as the threshold of statistical significance.

Evidence of excess significance bias—we compared the observed number (*O*) of claimed statistically significant studies (*P* < 0.05) with the number of studies expected (*E*) to be statistically significant to assess the presence of excess significance bias (19) by using chi-square statistics (21). Two-tailed *P* < 0.10 was considered statistically significant. The expected number of statistically significant studies in each meta-analysis was calculated by summing the statistical power estimates for each study, using an algorithm from a non-central t distribution, and the relative risk estimate of the largest study (i.e., the smallest standard error) was set as the plausible effect size (22). The excess significance test was considered positive when *P* < 0.10 given that *O* > *E*.

Application of credibility ceiling—we used a 10% credibility ceiling as a sensitivity analysis tool to interpret the methodological limitations of observational studies, that is, the likelihood that a specifically directed effect cannot go beyond 10% regardless of the scale and quality of the observational study (23). Inter-study heterogeneity and summary relative risk estimates were re-estimated accordingly.

### Reviewing the Existing Evidence

We rated the claimed statistically significant (*P* < 0.05) associations between aspirin and cancer risk into four levels—strong, highly suggestive, suggestive, and weak according to the following criteria: *P* < 10^−6^, >1,000 cases, *P* < 0.05 of the largest study in the meta-analysis, I^2^ <50%, absence of small-study effects (*P* > 0.1 for Egger's test), the 95% PI excludes the null value, no excess significance bias (*P* > 0.1), and survived the 10% credibility ceiling (*P* < 0.05) for strong evidence; *P* < 10^−6^, >1,000 cases, *P* < 0.05 of the largest study in the meta-analysis for highly suggestive evidence; *P* < 10^−3^, >1,000 cases for suggestive evidence; and *P* < 0.05 for weak evidence (24).

All analyses were performed using STATA 12.0.

## Results

### Characteristics of the Systematic Reviews and Meta-Analyses Included

**Figure 1:**
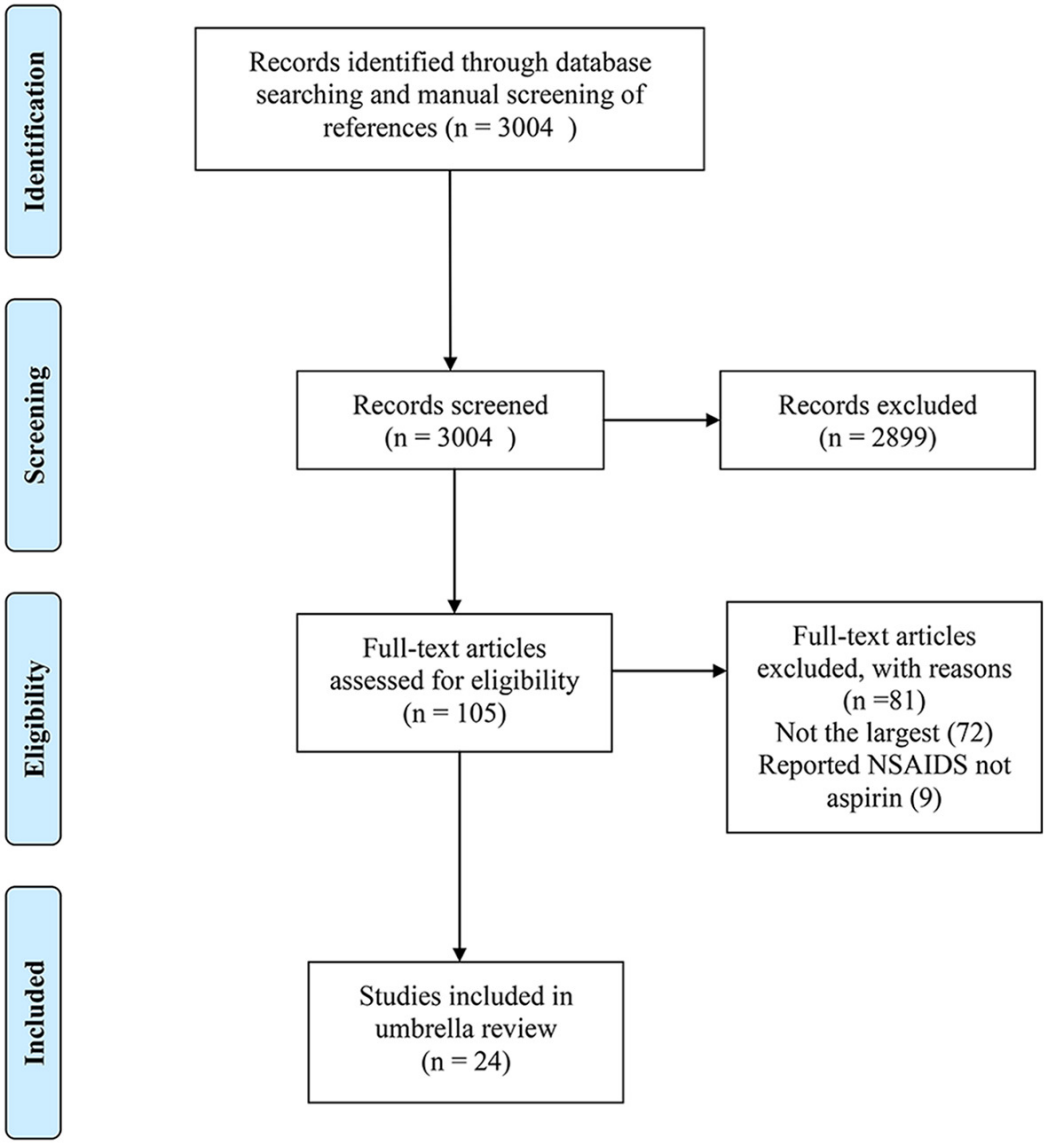
The flow diagram of study selection.

The literature search and manual screening of references identified 3,004 studies, of which 105 studies survived title and abstract screening, and 24 of these met the inclusion criteria and were ultimately included after full-text review (11, 12, 25–46). The flowchart of study selection is shown in Figure 1. The included studies covered 14 major anatomical sites, 27 different associations between aspirin and cancer risk, over 1.5 million cases and ~29 million subjects. Considering the well-established role of aspirin use in the prevention of CRC or colorectal adenoma for overall population, we included the study by Burr et al. (46) and the other by Veettil et al. (33) to explore the effect of aspirin on reducing CRC risk in subjects with inflammatory bowel disease (IBD) and on preventing colorectal adenoma in subjects with history of CRC or colorectal adenoma. The characteristics of the 27 associations are shown in Table 1. Data of the 589 individual studies from the 24 systematic reviews are available in Supplementary Material S1.

**Table 1:** Characteristics of the associations in the included systematic reviews and meta-analyses.

**Study**	**Association between aspirin and the risk of**	**Total studies**	**No of cases/population**	**Summary relative risk estimate (95% CI)**
Qiao et al. (38)^a^	Overall cancer	309	737359/15642135	0.89 (0.87–0.91)
Zhang et al. (28)^a^	Bladder cancer	11	8422/797725	1.02 (0.91–1.04)
Liu et al. (31)^a^	Brain tumor	8	13756/490663	1.01 (0.84–1.21)
Zhong et al. (26)^a^	Breast cancer	32	66531^#^/1334046^#^	0.90 (0.86–0.94)
Veettil et al. (33)^a^	Colorectal adenoma (recurrent)	5	1008/3958	0.82 (0.72–0.94)
Veettil et al. (33)^a^	Colorectal advanced adenoma (recurrent)	5	263/3213	0.70 (0.55–0.89)
Burr et al. (46)^a^	Colorectal cancer (in subjects with inflammatory bowel disease)	3	18^#^/86^#^	0.74 (0.26–2.08)
Zhang et al. (11)^a^	Esophageal adenocarcinoma (in subjects with Barrett's esophagus)	4	93^#^/1160^#^	0.63 (0.43–0.94)
Sivarasan et al. (35)^b^	Esophageal adenocarcinoma	9	2969/240699	0.67 (0.53–0.86)
Sun et al. (34)^b^	Esophageal squamous cell carcinoma	7	1026/18109	0.50 (0.39–0.63)
Huang et al. (44)^a^	Gastric cancer	11	3991^#^/153737^#^	0.72 (0.58–0.90)
Huang et al. (44)^a^	Non-cardia gastric cancer	7	1696/485340	0.64 (0.53–0.78)
Verdoodt et al. (32)^a^	Gynecological cancer (endometrial cancer)	13	11064/557597	0.93 (0.88–0.98)
Zhang et al. (29)^a^	Gynecological cancer (ovarian cancer)	22	14581/498700	0.89 (0.83–0.96)
Shi et al. (37)^a^	Head and neck cancer	20	2555/970276	0.87 (0.79–0.96)
Lee et al. (42)^a^	Hematological cancer (multiple myeloma)	3	605/331190	0.90 (0.58–1.39)
Ye et al. (30)^a^	Hematological cancer (non-hodgkin lymphoma)	12	3882/448435	1.02 (0.89–1.17)
Shoenfeld et al. (36)^a^	Liver cancer	5	478140/1386859	0.77 (0.58–1.02)
Hochmuth et al. (12)^a^	Lung cancer	20	15734/549760	0.86 (0.79–0.95)
Zhang et al. (27)^b^	Pancreatic cancer	8	2318/123594	0.77 (0.62–0.96)
Cui et al. (45)^b^	Pancreatic cancer (high dose aspirin)	8	3282/1129313	0.88 (0.76–1.01)
Cui et al. (45)^b^	Pancreatic cancer (low dose aspirin)	8	4985/1177556	0.99 (0.91–1.07)
Huang et al. (43)^a^	Prostate cancer	22	31858/509545	0.90 (0.85–0.95)
Muranushi et al. (40)^a^	Skin cancer (Basal cell carcinoma)	8	85613/305088	0.95 (0.91–0.99)
Muranushi et al. (39)^a^	Skin cancer (cutaneous squamous cell carcinoma)	6	4663/117489	0.88 (0.75–1.02)
Zhu et al. (25)^a^	Skin cancer	13	25764/893531	0.94 (0.90–0.99)
Li et al. (41)^a^	Skin cancer (melanoma)	10	7831/425858	0.97 (0.86–1.08)

*CI, confidence interval*.

a Reported odds ratio (RR);

b Reported risk ratio (OR);

# *Contain missing values*.

### Methodological Quality Assessment Results

All 24 systematic reviews had one or more critical flaws [usually in 7 (19/24, 79.2%) and 13 (23/24, 95.8%)] and several non-critical flaws [usually in items 3 (22/24, 91.7%), 10 (24/24, 100%), and 12 (23/24, 95.8%)] and were considered to have critically low methodological quality. The results of assessment and the rating criteria are shown in Supplementary Table S1.

### Summary Effect Size

The meta-analyses of the 27 associations were re-performed using a random-effect model. Two associations, including the associations between aspirin and the risk of overall cancer or esophageal squamous cell carcinoma, revealed a stringent statistical significance (*P* < 10^−6^) (Table 2 and Supplementary Table S2). The association between aspirin and the risk of breast cancer, non-cardia gastric cancer, or prostate cancer reached *P* < 10^−3^. The remaining 23 associations presented either *P* < 0.05 (13) or > 0.05 (10). The associations that reached statistical significance (17 in total) indicated potential preventive effects of aspirin on overall cancer, breast cancer, recurrent colorectal adenoma, recurrent advanced colorectal adenoma, esophageal adenocarcinoma in subjects with Barrett's esophagus, esophageal adenocarcinoma, esophageal squamous cell carcinoma, gastric cancer, non-cardia gastric cancer, endometrial cancer, ovarian cancer, head and neck cancer, lung cancer, pancreatic cancer, prostate cancer, overall skin cancer, or basal cell carcinoma.

**Table 2:** Evidence-rating results based on the results of statistical analyses of the 27 associations.

**Study**	**Association between aspirin and the risk of**	Summary relative risk estimate (random-effect *P*)^*^	**Cases >1000**	**Largest study relative risk estimate *P* < 0.05**	**I^**2**^ <50%**	**Small study effects**	**95% prediction interval exclude the null value**	**Excess significance**	**10% credibility ceiling survival**
Associations supported by strong evidence (1)									
Sun et al. (34)	Esophageal squamous cell carcinoma	+++	+	+	+	–	+	–	+
Associations supported by highly suggestive evidence (1)									
Qiao et al. (38)	Overall cancer	+++	+	+	–	+	–	+	+
Associations supported by suggestive evidence (3)									
Huang et al. (44)	Non-cardia gastric cancer	++	+	+	–	–	–	–	–
Zhong et al. (26)	Breast cancer	++	+	+	–	+	–	+	+
Huang et al. (43)	Prostate cancer	++	+	–	–	–	–	+	+
Associations supported by weak evidence (12)									
Zhang et al. (29)	Gynecological cancer (ovarian cancer)	+	+	–	+	+	–	–	+
Sivarasan et al. (35)	Esophageal adenocarcinoma	+	+	–	–	–	–	+	+
Hochmuth et al. (12)	Lung cancer	+	+	+	–	–	–	–	+
Huang et al. (44)	Gastric cancer	+	+	–	–	+	–	–	+
Veettil et al. (33)	Colorectal advanced adenoma (recurrent)	+	–	–	+	–	–	–	+
Veettil et al. (33)	Colorectal adenoma (recurrent)	+	+	–	+	–	–	+	+
Shi et al. (37)	Head and neck cancer	+	+	–	–	+	–	+	–
Verdoodt et al. (32)	Gynecological cancer (endometrial cancer)	+	+	–	+	+	+	–	+
Zhang et al. (27)	Pancreatic cancer	+	+	–	–	–	–	+	–
Zhu et al. (25)	Skin cancer	+	+	–	+	–	–	+	+
Zhang et al. (11)	Esophageal adenocarcinoma (in subjects with Barrett's esophagus)	+	–	–	+	–	–	–	+
Muranushi et al. (40)	Skin cancer (Basal cell carcinoma)	+	+	–	–	–	–	+	+
Associations supported by not suggestive evidence (10)									
Cui et al. (45)	Pancreatic cancer (high dose aspirin)	–	+	–	+	+	–	–	–
Shoenfeld et al. (36)	Liver cancer	–	+	+	–	–	–	–	–
Muranushi et al. (39)	Skin cancer (cutaneous squamous cell carcinoma)	–	+	+	–	–	–	–	–
Li et al. (41)	Skin cancer (melanoma)	–	+	+	–	–	–	–	–
Burr et al. (46)	Colorectal cancer (in subjects with inflammatory bowel disease)	–	–	–	–	–	–	–	–
Lee et al. (42)	Hematological cancer (multiple myeloma)	–	–	+	–	–	–	–	–
Cui et al. (45)	Pancreatic cancer (low dose aspirin)	–	+	–	+	–	–	–	–
Zhang et al. (28)	Bladder cancer	–	+	–	+	–	–	–	–
Ye et al. (30)	Hematological cancer (non-Hodgkin lymphoma)	–	+	–	+	–	–	–	–
Liu et al. (31)	Brain tumor	–	+	–	–	–	–	–	–

* *P-value calculated using random-effect model: +++, P < 10^−6^; ++, P < 10^−3^; +, P < 0.05; –, P > 0.05. For other items, + = yes, – = no*.

### Heterogeneity

Sixteen of the 27 (59%) associations presented substantial heterogeneity (>50%). The 95% PI was also calculated to further assess inter-study heterogeneity. Only the 95% PIs of two associations—aspirin and the risk of esophageal squamous cell carcinoma or endometrial cancer—excluded the null value (Table 2 and Supplementary Table S2).

### Small-Study Effects

Small study effects were detected in seven associations: aspirin and the risk of overall cancer, breast cancer, head and neck cancer, ovarian cancer, endometrial cancer, or gastric cancer at *P* < 0.1 for Egger's test (Table 2 and Supplementary Table S2). However, 15 of the 27 (56%) associations included fewer than 10 studies and were inadequate to enable Egger's test to detect small-study effects.

### Excess Significance

The excess significance test was positive (*P* < 0.1 AND *O* > *E*) in nine associations: between aspirin and the risk of overall cancer, breast cancer, esophageal adenocarcinoma, recurrent colorectal adenoma, head and neck cancer, pancreatic cancer, overall skin cancer, or basal cell carcinoma (Table 2 and Supplementary Table S2).

### Credibility Ceiling

Fourteen of the 27 associations survived the 10% credibility ceiling, including the association between aspirin and the risk of overall cancer, breast cancer, recurrent colorectal adenoma, recurrent advanced colorectal adenoma, esophageal adenocarcinoma in subjects with Barrett's esophagus, esophageal adenocarcinoma, esophageal squamous cell carcinoma, gastric cancer, endometrial cancer, ovarian cancer, lung cancer, prostate cancer, overall skin cancer, or basal cell carcinoma (Table 2 and Supplementary Table S2).

### Robustness of Evidence

Out of 27 associations between aspirin and cancer risk, only one association (aspirin and esophageal squamous cell carcinoma) was supported by strong evidence. The association between aspirin and overall cancer risk was supported by highly suggestive evidence. The evidence supporting the association between aspirin and the risk of breast cancer, non-cardia gastric cancer, or prostate cancer was considered to be highly suggestive (Table 2). The remaining 22 associations were supported by weak (12) or not suggestive evidence (10). The detailed results of the analyses on which the evidence rating was based are shown in Supplementary Table S2.

## Discussion

### Main Findings and Interpretation in Light of Evidence

Of note, aspirin was recommended for the prevention of CRC by the USPSTF in 2016, and the main focus of this umbrella review is on the systematic reviews exploring the potential preventive role of aspirin in other cancers or in CRC or colorectal adenoma but in specified populations, such as in subjects with IBD or with history of CRC or colorectal adenoma, so that new indications of aspirin for more cancers may be made which will help protect more people from more cancers. We included 24 systematic reviews and meta-analyses that covered 14 major anatomical sites, 27 different associations, 589 primary studies, more than 29 million subjects, and more than 1.5 million cases. We also evaluated the methodological quality of the 24 studies and evaluated the validity of the evidence supporting the 27 associations identified based on assessment results of the aforementioned analyses.

Only one association (aspirin and esophageal squamous cell carcinoma) was supported by strong evidence as most of the associations did not reach a stringent *P-*value (10^−6^) and presented substantial heterogeneity (I^2^ > 50% or 95% PI did not exclude the null value). According to our analyses, aspirin use brings a stringently significant 50% reduction in the incidence of esophageal squamous cell carcinoma [RR: 0.50 (95% CI, 0.39–0.63)] with great credibility. Of note, the preventive effect of aspirin on esophageal adenoma in subjects with or without Barrett's esophagus (both deemed weak evidences) is less certain due to significant heterogeneity and hints of bias. Consequently, researchers and policy makers should pay more attention to the potential merit of repositioning aspirin to prevent esophageal squamous cell carcinoma and meanwhile, be cautious when dealing with those with esophageal adenoma unless future studies provides more robust evidence. The association between aspirin and overall cancer incidences was supported by highly-suggestive evidence, which demonstrated an overall anti-cancer activity of aspirin [RR: 0.89 (95% CI, 0.87–0.91)]. However, it is hard for clinicians and decision makers to interpret these results as substantial heterogeneity exists which leads to considerable uncertainties on whether aspirin works to protect people from a specific cancer. The associations between aspirin and the incidences of breast cancer, non-cardia gastric cancer, or prostate cancer were supported by suggestive evidence, indicating a less certain but still likely preventive role of aspirin in these cancers. As for the associations supported by weak evidences, considering that these associations present statistically significant results, decision-makers should be cautious when interpreting these results and do not arbitrarily refer to these associations when making decisions. When it comes to the associations supported by not suggestive evidences, the results are not statistically significant in the first place, thus are automatically not suggested to be referred to during decision-making. However, this does not necessarily mean that no further study is needed especially for the associations covering < 1,000 cases. The possible explanations for the statistically insignificant results can be various, including the insufficient sample size and the retrospective nature of the study designs of the studies included in these meta-analyses supported by “not suggested” evidences. In fact, associations supported by less certain evidences can be of merit under certain circumstances. For example, compared with the strong effect of aspirin on reducing CRC risk in overall population, the weak association between aspirin use and CRC risk in subjects with IBD can be plausible supporting material for the conclusion that IBD increases the risk of CRC, which has been reported in substantial relevant studies. Similarly, the statistically insignificant results presented in the associations between aspirin use and risk of recurrent (advanced) adenomas somewhat coincide with the “adenoma-carcinoma sequence” in the development of CRC, indicating that subjects who have already suffered this sequence cannot benefit from preventive aspirin use. Moreover, the discrepancy between the evidences supporting associations on esophageal squamous cell carcinoma or adenocarcinoma may facilitate further studies on the mechanism of the discrepant efficacy of aspirin on these two different histologic types of esophageal cancer.

### Strengths and Limitations

This umbrella review is the first and the most comprehensive systematic review of systematic reviews and meta-analyses on the potential preventive role of aspirin in various cancers. The robustness and the validity of a total of 27 associations were strictly rated based on the assessment results of a series of statistical analyses. The methodological qualities of the systematic reviews included were assessed using AMSTAR 2.0 checklist which is a major update of the former version AMSTAR. The superiority of AMSTAR 2.0 compared with AMSTAR was described elsewhere (16). The results of the methodological quality assessment indicated that the robustness of the 24 included studies was critically low. Most of the included studies did not register the research protocol in registry websites (item 2), did not justify the exclusion of potentially eligible studies (item 7), and did not account for the risk of bias (RoB) in individual studies when interpreting or discussing the results of systematic reviews and meta-analyses (item 13), which are all critical domains in AMSTAR 2.0, and these limitations contributed to the negative ratings. Moreover, the included studies should consider the study design of individual studies (item 3), report the source of funding for the primary studies (item 10), and evaluate the potential impact of the RoB of individual studies on the results of quantitative or qualitative syntheses (item 12) as too many non-critical items lower the rating. Previously published umbrella reviews assessed studies using the 11-item AMSTAR but did not interpret the results thoroughly by calculating overall scores and failing to establish a criterion using AMSTAR to rate systematic reviews according to individual scores (24). Compared with the blurred assessment results of AMSTAR, AMSTAR 2.0 managed to scrape the tip of the iceberg in terms of the low quality of existing systematic reviews and meta-analyses, focusing on the association between aspirin use and cancer. Therefore, we should interpret the current evidence in this field with caution.

There are several limitations worth mentioning. First, as aforementioned, the methodological quality of all systematic reviews included was considered to be critically low according to the assessment using the AMSTAR 2.0 checklist. Second, further analyses were not possible because data on dose, frequency, or duration of aspirin use reported in these studies were lacking, which makes it more complicated for public health policy-makers to recommend prophylactic aspirin use in guidelines. Furthermore, over half of the associations (15) failed to include sufficient studies (at least 10) to enable excess significance tests and Egger's tests to identify the origin of biases (20).

## Conclusion

The association between aspirin and a reduced risk of esophageal squamous cell carcinoma is supported by strong evidence, researchers and policy makers should pay more attention to the potential merit of repositioning aspirin to prevent esophageal squamous cell carcinoma.

## Data Availability Statement

All datasets generated for this study are included in the article/Supplementary Material.

## Author Contributions

ZWa, YS, and XZ conceived and designed the study. YS and XZ performed the literature search, acquired, and collated the data, which were analyzed by XZ, PG, CZ, JS, ZWu, and ZG. ZWa was guarantor. ZWa attests that all listed authors meet authorship criteria and that no others meeting the criteria have been omitted. All authors drafted and critically revised the manuscript for important intellectual content. All authors gave final approval of the version to be published and contributed to the manuscript.

### Conflict of Interest

The authors declare that the research was conducted in the absence of any commercial or financial relationships that could be construed as a potential conflict of interest.

## Supplementary Material

Supplementary Table S1 The detailed results of methodological quality assessment.

Supplementary Table S2 Results of statistical analyses for the evidence rating of the 27 associations.

Supplementary Table S3 The dataset generated for analysis.
