# Supplementary material for: Aspirin and Its Potential Preventive Role in Cancer: An Umbrella Review
Source: Front Endocrinol (Lausanne). 2020 Jan 23;11:3. doi: 10.3389/fendo.2020.00003 (PMC6989406; doi:10.3389/fendo.2020.00003)
Supplement: Supplementary Table S2 — Results of statistical analyses for the evidence rating of the 27 associations. [file Table_2.DOCX]

**Supplementary Table S2. Results of statistical analyses for the evidence rating of the 27 associations**

| Study | Association between aspirin and the risk of | Summary relative risk (random effect) | | Cases | Largest study | I^2^  (95% CI) | *P* value for Egger’s test | 95% prediction interval | Excess significance test | | 10% credibility ceiling  (*P* value) |
| --- | --- | --- | --- | --- | --- | --- | --- | --- | --- | --- | --- |
|  |  | Estimate | *P*  value | *N* | Relative risk estimate (95% CI) |  |  |  | *O/E* | *P*  value |  |
| Qiao et al, 2018^1^ | Overall cancer | 0.89  (0.87-0.91) | 8.71*10^-25^ | 737359 | 0.97  (0.94-0.99) | 80%  (78%-82%) | <0.001 | (0.68-1.17) | 99/30.4 | 3.98*10^-39^ | 1.1*10^-5^ |
| Zhang et al, 2013^1^ | Bladder cancer | 1.02  (0.91-1.14) | 0.781 | 8422 | 1.04  (0.94-1.15) | 49%  (0%-73%) | 0.686 | (0.75-1.37) | 2/0.9 | 0.226 | 0.467 |
| Liu et al, 2014^1^ | Brain tumor | 1.01  (0.84-1.21) | 0.946 | 13756 | 1.09  (0.99-1.20) | 79%  (52%-88%) | 0.644 | (0.57-1.77) | 3/2.8 | 0.882 | 0.750 |
| Zhong et al, 2015^1^ | Breast cancer | 0.90  (0.86-0.94) | 1.72*10^-5^ | 66745^#^ | 0.94  (0.90-0.99) | 76%  (66%-82%) | 0.012 | (0.72-1.13) | 15/7.0 | 0.003 | 0.052 |
| Veetil et al, 2017^1^ | Colorectal adenoma (recurrent) | 0.82  (0.72-0.94) | 0.003 | 1008 | 0.88  (0.77-1.02) | 30%  (0%-74%) | 0.557 | (0.59-1.15) | 2/0.7 | 0.094 | 0.039 |
| Veetil et al, 2017^1^ | Colorectal advanced adenoma (recurrent) | 0.70  (0.55-0.89) | 0.003 | 263 | 0.71  (0.50-1.01) | 0%  (0%-64%) | 0.532 | (0.48-1.03) | 1/1.2 | 0.834 | 0.099 |
| Burr et al 2016^1^ | Colorectal cancer (in subjects with inflammatory bowel disease) | 0.74  (0.26-2.08) | 0.572 | 18^#^ | 1.52  (0.70-3.25) | 67%  (0%-88%) | 0.602 | (0-78317) | 1/1.2 | 0.813 | 0.934 |
| Zhang et al, 2014^1^ | Esophageal adenocarcinoma (in subjects with Barrett’s esophagus) | 0.63  (0.43-0.94) | 0.024 | 93^#^ | 0.66  (0.37-1.17) | 0%  (0%-68%) | 0.806 | (0.26-1.51) | 0/1.7 | 0.086 | 0.074 |
| Sivarasan et al, 2013^2^ | Esophageal adenocarcinoma | 0.67  (0.53-0.86) | 1.29*10^-3^ | 2969 | 0.88  (0.70-1.10) | 75%  (43%-85%) | 0.105 | (0.30-1.50) | 5/1.8 | 0.008 | 0.045 |
| Sun et al, 2011^2^ | Esophageal squamous cell carcinoma | 0.50  (0.39-0.63) | 3.13*10^-9^ | 1026 | 0.46  (0.30-0.73) | 0%  (0%-58%) | 0.484 | (0.37-0.67) | 5/6.5 | 0.028 | 0.006 |
| Huang et al, 2017^1^ | Gastric cancer | 0.72  (0.58-0.90) | 0.003 | 3991^#^ | 1.15  (0.98-1.36) | 72%  (41%-83%) | 0.089 | (0.37-1.43) | 4/3.4 | 0.695 | 0.069 |
| Huang et al, 2017^1^ | Non-cardia gastric cancer | 0.64  (0.53-0.78) | 9.60*10^-6^ | 1696 | 0.73  (0.61-0.89) | 69%  (7%-84%) | 0.105 | (0.35-1.18) | 5/4.3 | 0.587 | 0.010 |
| Verdoodt et al, 2016^1^ | Gynecological cancer (endometrial cancer) | 0.93  (0.88-0.98) | 0.008 | 11064 | 0.97  (0.89-1.05) | 0%  (0%-49%) | 0.073 | (0.87-0.99) | 1/1.0 | 0.958 | 0.042 |
| Zhang et al, 2016^1^ | Gynecological cancer (ovarian cancer) | 0.89  (0.83-0.96) | 1.21*10^-3^ | 14581 | 0.94  (0.85-1.04) | 23%  (0%-54%) | 0.005 | (0.75-1.06) | 3/2.6 | 0.792 | 0.030 |
| Shi et al, 2017^1^ | Head and neck cancer | 0.87  (0.79-0.96) | 0.008 | 2555 | 1.02  (0.88-1.18) | 66%  (41%-78%) | 0.006 | (0.59-1.29) | 5/1.0 | 4.06*10^-5^ | 0.134 |
| Lee et al, 2017^1^ | Hematological cancer (multiple myeloma) | 0.90  (0.58-1.39) | 0.642 | 605 | 0.63  (0.41-0.96) | 51%  (0%-85%) | 0.271 | (0.01-79.10) | 1/2.5 | 0.020 | 0.898 |
| Ye et al, 2015^1^ | Hematological cancer (non-hodgkin lymphoma) | 1.02  (0.89-1.17) | 0.811 | 3882 | 0.93  (0.74-1.16) | 27%  (0%-62%) | 0.464 | (0.74-1.39) | 1/1.2 | 0.847 | 0.935 |
| Shoenfeld et al, 2017^1^ | Liver cancer | 0.77  (0.58-1.02) | 0.070 | 478140 | 0.63  (0.50-0.79) | 78%  (23%-89%) | 0.783 | (0.28-2.09) | 2/3.7 | 0.288 | 0.461 |
| Hochmuth et al, 2016^1^ | Lung cancer | 0.86  (0.79-0.95) | 0.002 | 15734 | 1.11  (1.02-1.20) | 75%  (59%-83%) | <0.001 | (0.61-1.24) | 7/5.3 | 0.389 | 0.821 |
| Zhang et al, 2015^2^ | Pancreatic cancer | 0.77  (0.62-0.96) | 0.019 | 2318 | 0.95  (0.81-1.12) | 74%  (36%-86%) | 0.413 | (0.39-1.52) | 4/0.5 | 3.19*10^-7^ | 0.359 |
| Cui et al, 2014^2^ | Pancreatic cancer (high dose aspirin) | 0.88  (0.76-1.01) | 0.069 | 3282 | 0.97  (0.86-1.09) | 13%  (0%-62%) | 0.076 | (0.68-1.14) | 1/0.5 | 0.465 | 0.222 |
| Cui et al, 2014^2^ | Pancreatic cancer (low dose aspirin) | 0.99  (0.91-1.07) | 0.732 | 4985 | 0.98  (0.92-1.05) | 16%  (0%-63%) | 0.570 | (0.84-1.15) | 0/0.5 | 0.465 | 0.734 |
| Huang et al, 2014^1^ | Prostate cancer | 0.90  (0.85-0.95) | 1.85*10^-4^ | 31858 | 1.01  (0.95-1.07) | 55%  (18%-71%) | 0.169 | (0.76-1.08) | 9/1.2 | 2.43*10^-13^ | 0.065 |
| Muranushi et al, 2016^1^ | Skin cancer (Basal cell carcinoma) | 0.95  (0.91-0.99) | 0.030 | 85613 | 0.98  (0.95-1.02) | 55%  (0%-78%) | 0.012 | (0.85-1.07) | 1/1.1 | 0.918 | 0058 |
| Muranushi et al, 2015^1^ | Skin cancer (cutaneous squamous cell carcinoma) | 0.88  (0.75-1.02) | 0.098 | 4663 | 0.86  (0.76-0.98) | 64%  (0%-83%) | 0.818 | (0.55-1.39) | 2/2.7 | 0.566 | 0.198 |
| Zhu et al, 2015^1^ | Skin cancer | 0.94  (0.90-0.99) | 0.020 | 25764 | 0.99  (0.96-1.03) | 48%  (0%-71%) | 0.195 | (0.83-1.07) | 4/0.7 | 5.01*10^-5^ | 0.091 |
| Li et al, 2013^1^ | Skin cancer (melanoma) | 0.97  (0.86-1.08) | 0.555 | 7831 | 0.89  (0.80-0.98) | 67%  (21%-81%) | 0.672 | (0.68-1.37) | 4/3.3 | 0.638 | 0.636 |

1 Reported odds ratio (RR);

2 Reported risk ratio (OR);

# contain missing values.

*N*= number; *O*= the number of observed statistically significant studies; *E*= the number of expected statistically significant studies
